# Spatial Variability of Soil and Plant Water Status and Their Cascading Effects on Grapevine Physiology Are Linked to Berry and Wine Chemistry

**DOI:** 10.3389/fpls.2020.00790

**Published:** 2020-06-23

**Authors:** Runze Yu, Luca Brillante, Johann Martínez-Lüscher, Sahap Kaan Kurtural

**Affiliations:** Department of Viticulture and Enology, University of California, Davis, Davis, CA, United States

**Keywords:** viticulture, plant water status, soil electrical conductivity, spatial variability, flavonoids, wine

## Abstract

The relationships between differences in plant water status, induced by spatial variability in soil texture, and the changes in berry and wine composition were investigated in an irrigated Cabernet Sauvignon (*Vitis vinefera* L.) vineyard for 2 years. A stratified and an equidistant grid were overlaid on the vineyard to characterize the soil texture by proximal sensing, soil sampling, and grapevine physiological and berry chemical development. Based on the mid-day stem water potential (Ψ_*stem*_) integrals, the vineyard was divided into two functional homogenous zones: Zone 1 with higher water stress and Zone 2 with lower water. Zone 1 consistently had lower Ψ_*stem*_, net carbon assimilation, and stomatal conductance in both years. Berry weight and titratable acidity were lower in Zone 1 at harvest. Zone 2 reached 26 and 24°Bx total soluble solids (TSS) at harvest in Years 1 and 2, respectively, with higher TSS values of 30 and 27°Bx in Zone 1. Ravaz index did not vary spatially. Fruits were harvested differentially in both years and vinified separately from the two zones. In Year 1, all berry skin anthocyanin derivatives, tri-, di- hydroxylated, and total anthocyanins concentrations were higher in Zone 2. However, in Year 2, only malvidin, tri-hydroxylated, and total anthocyanins were higher in Zone 1. There were no differences in wine flavonoids in Year 2 when harvest commenced earlier. In both years, Ψ_*stem*_, berry weight, and TSS were directly related to soil bulk electrical conductivity (EC). Our results indicated vineyard variability stemmed from soil texture that affected long-term plant water status which does not affect spatial variability of Ravaz Index. In conclusion, our work provides fundamental knowledge about the applicability of soil bulk EC sensing in the vineyards, and its potential directional utilization by connecting proximal soil sensing to spatial distribution of whole-plant physiological performance together with berry and wine chemistry.

## Introduction

There is natural spatial variability present in vineyards due to the variations in soil characteristics and topography (Brillante et al., 2016a). Soil characteristics are too complex to be thoroughly surveyed effortlessly. With traditional destructive methods, it is difficult to obtain enough comprehensive information from the soil pits at the field scale. These soil characteristics may directly affect the water availability for grapevines, which eventually determine the physiological performance of the plants (Brillante et al., 2015, 2016a). However, there is no variable management practices currently available to accommodate the natural spatial variability. Thus, the spatial variability derived from vineyard soils will inevitably be expressed in the whole plant physiology at the cost of homogeneity of vineyard productivity and quality. We previously reported the spatial variation of mid-day stem water potential affecting grapevine carbon assimilation and stomatal conductance of grapevine (Brillante et al., 2017; Yu and Kurtural, 2020). The resultant variations in whole-plant physiology were associated to flavonoid composition and concentration at the farm gate. However, there is a lack of information about the effects on the chemical composition in the final wine, which would ultimately determine wine quality as perceived by consumers.

Georeferenced proximal sensing tools can capture the spatial and temporal variability in vineyards, making it possible to supervise and manage variations at the field scale (Bramley et al., 2011c; Matese et al., 2015). Previous studies showed that soil bulk electrical conductivity (EC) may be used to evaluate many soil attributes, including soil moisture content, salinity, and texture (Brillante et al., 2014; Su et al., 2014). Soil electromagnetic induction (EMI) sensing has been used in precision agriculture to acquire soil bulk EC at the field scale due to its non-invasive and prompt attributes (Bramley et al., 2011a; Rodríguez-Pérez et al., 2011). Although research had been conducted on the relationships between soil electrical properties with plant water status, they were mostly point measurements and the results were rarely interpolated to whole fields. There were only a few studies that investigated the EMI sensing and soil-plant water relationships over a vineyard (Bonfante et al., 2015). Previous research suggested that the connection between soil water content and soil bulk EC could have relied on specific soil profiles, and needed to include soil physical and chemical properties to complete this connection (Brillante et al., 2014, 2016a). Nevertheless, there is evidence that soil bulk EC may still be useful not only to identify the variability in soil, but also in the plant response affected by vineyard soils such as yield, plant physiology, and grape berry chemistry (Bramley et al., 2011a; Tagarakis et al., 2013).

Plant available water is a determinant factor on grapevine physiology, together with nitrogen availability in semi-arid regions (Smart and Coombe, 1983). Wine grapes are usually grown under a moderate degree of water deficits as yields were optimized at 80% of crop evapotranspiration demand with sustained deficit irrigation (Williams, 2012). Water deficits would limit leaf stomatal conductance and carbon assimilation rate that sustain grapevines’ vegetative and reproductive growth and development (Escalona et al., 2015). When grapevines are under water deficits, carbohydrates repartitioned into the smaller berries would enhance berry soluble solids content (Escalona et al., 2015). Sucrose and fructose, which are the major components of total soluble solids (TSS) in grape berry, can act as a signaling factor to stimulate anthocyanin accumulation (Dai et al., 2014). The effects on grapevine physiology and berry composition also depend on the phenological stages they occur and how severe and prolonged the water deficits are (Intrigliolo and Castel, 2010).

Flavonoids are the most critical compounds dictating many qualitative traits in both grape berries and wine (Lorrain et al., 2013). The variations in environmental factors could alter the concentration and biosynthesis of flavonoids and can be extrapolated spatially within the same vineyard, including water deficits (Castellarin et al., 2007b), solar radiation (Martínez-Lüscher et al., 2019), and air temperature (Spayd et al., 2002). Among flavonoid compounds, anthocyanins are responsible for the color of berry skin as well as wine (Intrigliolo and Castel, 2010). Moderate water deficits during growing season can increase anthocyanin concentration in berry skin and wine (Cortell et al., 2007). However, water deficits can impair plant temperature regulation through evaporative cooling (Tombesi et al., 2015). They may also inhibit berry growth by limiting berry size and altering berry skin weight (Castellarin et al., 2007a; Santesteban et al., 2011). Thus, in some cases it may be uncertain if water deficit promotes anthocyanins biosynthesis or reduces berry growth, or contributes to anthocyanin degradation (Petrussa et al., 2013). Applying water deficit on grapevines can contribute to greater proportion in tri-hydroxylated over di-hydroxylated anthocyanins due to the up-regulation of F3′5′H (Castellarin et al., 2007b; Martínez-Lüscher et al., 2014). Another major class in flavonoids, proanthocyanidins, are polymers of flavan-3-ol monomers and they contributes mainly toward astringency (tactile sensation) or bitterness (taste) in wine (Gonzalo-Diago et al., 2013). Compared to anthocyanins, water deficits showed mild effects on proanthocyanidins (Bucchetti et al., 2011). However, water deficits with great severity can still alter the concentration and composition of proanthocyanidins in both berries and wine (Ollé et al., 2011).

Selective harvest is one of the targeted management strategies to minimize the spatial variation in berry chemistry in vineyards (Scarlett et al., 2014). By differentially harvesting or segregating the fruits into batches prior to vinification, the berry composition can be artificially set at a more uniform stage with minimal variations (Bramley et al., 2011b). In our previous work, we reported the use of plant water status to determine the spatial variation of grape berry flavonoids (Brillante et al., 2017). The goal of this study was to deduce if the spatial variability of soil bulk EC and differences in soil texture can be related to plant physiology and grape and wine composition. The specific objective of the study was to determine if the spatial variability of proximally sensed vineyard soil bulk EC would affect plant water status, and if this relation would affect leaf gas exchange, components of yield, berry composition, and flavonoids in both berries and wine.

## Materials and Methods

### Vineyard Site, Plant Materials, and Weather

**FIGURE 1:**
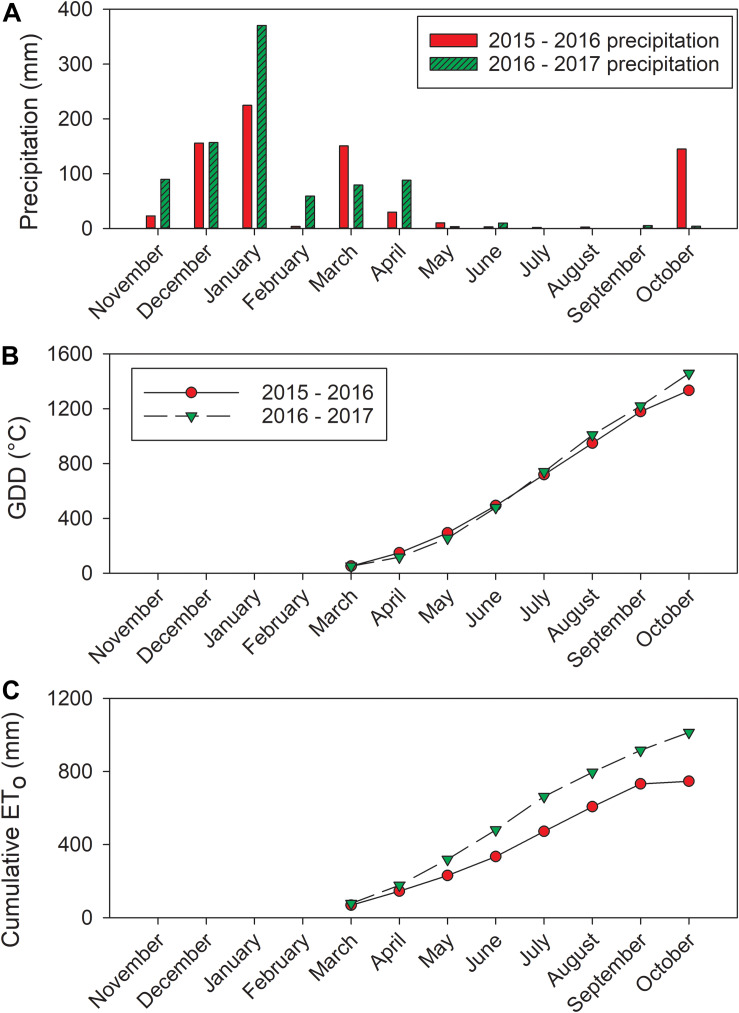
Weather data acquired from California Irrigation Management Information System (CIMIS) station (#86, Santa Rosa, CA). **(A)** Precipitation, **(B)** calculated GDD, and **(C)** cumulative ET_*o*_.

The study was conducted in a commercial vineyard in 2016 and 2017 with Cabernet Sauvignon (*Vitis vinifera* L.) grapevines grafted on 110R (*Vitis berlandieri* Planch. × *Vitis rupestris* Scheele) located in Healdsburg, CA, United States. In this vineyard, grapevines were planted at 1.83 m × 3.35 m (vine × row). The grapevines were trained to a high-quadrilateral, horizontally split trellis with two bilateral cordons. They were spur pruned with two buds per spur, and seven spurs per meter of the cordon. Irrigation was applied uniformly with a drip irrigation system, starting at fruit-set to the end of veraison at 50% ET_*c*_. There were two emitters per grapevine, delivering 3.8 L⋅h^–1^ of water. Weather data was obtained from the California Irrigation Management Information System (CIMIS) station (#86, Santa Rosa, CA, United States) to measure precipitation, air temperature, and reference evapotranspiration (Figure 1).

### Experimental Design

An equidistant 33 m × 33 m grid with 35 experimental units was used for on-site measurements and berry samplings. Each experimental unit consisted of five plants. The locations of each central plant in these five plant experimental units were registered as the grid nodes with a GPS (Yuma 2, Trimble Inc., Sunnyvale, CA, United States), wirelessly connected to a Trimble Pro 6T DGNSS receiver (Trimble Inc., Sunnyvale, CA, United States).

### Vineyard Soil Property Assessment

Soil bulk EC was assessed with EM38 (Geonics Ltd., Mississauga, ON, Canada) in 2016 when the vineyard soil was at field capacity condition. Both vertical dipole mode and horizontal dipole mode were used to assess EC at two depths, including deep soil (0–1.50 m) and shallow soil (0–0.75 m). The instrument was calibrated according to manufacturer instructions. The device was placed on a PVC sled and driven through the vineyard with an all-terrain vehicle along the inter-rows. A distance of approximately 0.5 m from the vehicle to the device was maintained to avoid interference with the vehicle. A stratified grid was used to collect soil samples corresponding to the two depths at which we measured soil bulk EC. Soil texture was assessed according to the soil analysis method: hydrometer analysis (S – 14.10) in the North American Proficiency Testing (NAPT) program.

### Grapevine Physiology Assessments

Plant water status was assessed biweekly by midday stem water potential (Ψ_*stem*_) measurements. The measurements for Ψ_*stem*_ in 2016 were previously described in 2016. In 2017, Ψ_*stem*_ was assessed on 27 June, 13 July, 27 July, 8 August, 24 August, 8 September, and 19 September. The measurements were conducted at solar noon from 12:00 to 14:30 h. Three leaves from main shoot axes in the shade were selected and concealed in pinch-sealed Mylar^®^ bags for 2 h prior to the measurements. A pressure chamber (Model 615D, PMS Instrument Company, Albany, OR, United States) was used to take the measurements. To summarize the season-long plant water status, Ψ_*stem*_ integrals were calculated by using natural cubic splines, and then normalized by the number of days elapsed from the first measurement to the last.

Leaf gas exchange measurements were taken biweekly by using a portable infrared gas analyzer CIRAS-3 (PP Systems, Amesbury, MA, United States). The measurements for leaf gas exchange in 2016 were previously described in 2016. In 2017, leaf gas exchange was assessed on 13 July, 27 July, 8 August, and 24 August. The gas analyzer was set to a relative humidity of 40% and the reference CO_2_ concentration of 400 μmol CO_2_⋅mol^–1^. Three sun-exposed leaves from the main shoot axis were measured in each vine, and the three middle vines were selected in each experimental unit. Gas exchange measurements were taken when the sunlight was at saturation conditions in both years (average PAR_*i*_ = 1969 ± 135 μmol⋅m^–2^⋅s^–1^ in 2016, 1884 ± 165 μmol⋅m^–2^⋅s^–1^ in 2017).

Yield components were measured on a single harvest day in each season (5 October 2016 and 20 September 2017). The dates were chosen to follow the grower’s harvest schedule. The clusters from the three middle vines in each experimental unit were harvested, counted, and weighed. Cluster weight was calculated by dividing crop weight by cluster number. A total of 75 berries were randomly selected from the five vines in each experimental unit, and were separated into two subsets of 55 and 20 berries. The first set with 55 berries was used for berry composition analysis. The second set with 20 berries was for measuring berry skin mass and skin flavonoid contents. The average berry weight were assessed from the average weight of the total 75 berries. Pruning weight per vine was collected during the dormant season. Ravaz index was calculated as the ratio of the yield per vine and the pruning weight per vine.

### Berry Total Soluble Solids, pH, and Titratable Acidity

Berry samples were taken biweekly throughout each season. In 2016, berry wet chemistry was assessed on 15 July, 28 July, 11 August, 23 August, 1 September, 15 September, and 5 October. In 2017, berry wet chemistry was assessed on 13 July, 27 July, 8 August, 24 August, 7 September, and 20 September. Total soluble solids (TSS, measured as°Brix), pH, and titratable acidity (TA) were analyzed on the must. Berry TSS were measured by a digital refractometer (Atago PR-32, Bellevue, WA, United States). Must pH and TA (expressed as g of tartaric acid per L of must after titration to pH 8.3) were measured with an automated titrator (862 Compact TitroSampler, Metrohm, Switzerland).

### Extraction of Skin Flavonoid Compounds

Skins were manually peeled from the 20 berries with a scalpel, and lyophilized by a freeze-drier (Triad Freeze-Dry System, Labconco, Kansas City, MO, United States). Skin tissues were then powderized with a mixing mill (MM400, Retsch, Mammelzen, Germany). For anthocyanin analysis, 50 mg of dry skin powder was weighed and extracted with 1 mL of methanol:water:7 M hydrochloric acid (70:29:1) solution at 4°C overnight. Extracts were centrifuged at 5,000 rpm for 10 min, the supernatants were filtered by PTFE membrane filters (diameter: 13 mm, pore size: 0.45 μm, VWR, Seattle, WA, United States), and transferred into high performance liquid chromatography system (HPLC) vials before injection.

### Berry and Wine Flavonoid Analysis

Skin anthocyanins were analyzed by a reversed-phase HPLC (Agilent model 1260, Agilent Technologies, Santa Clara, CA, United States) consisting of a vacuum degasser, an autosampler, a quaternary pump, and a diode array detector with a column heater. A C18 reversed-phase column (LiChrosphere 100 RP-18, 4 × 520 mm^2^, 5 μm particle size, Agilent Technologies, Santa Clara, CA, United States) was utilized for analyzing anthocyanins. The mobile phase flow rate was 0.5 mL⋅min^–1^, and two mobile phases were used, which included solvent A = 5.5% aqueous formic acid (v/v) and solvent B = 5.5% formic acid in acetonitrile (v/v). The HPLC flow gradient started with 91.5% A with 8.5% B; 87% A with 13% B at 25 min; 82% A with 18% B at 35 min; 62% A with 38% B at 70 mins; 50% A with 50% B at 70.01 min; 30% A with 70% B at 75 min; 91.5% A with 8.5% B from 75.01 min to 90 min. The column temperature was maintained at 25°C. Detection of anthocyanins was carried out by the diode array detector at 520 nm. A computer workstation with Agilent OpenLAB (Chemstation edition, version A.02.10) was used for chromatographic analysis.

Wine proanthocyanidin subunits were characterized by acid catalysis in the presence of excess phloroglucinol by reversed-phase HPLC (Agilent model 1100, Agilent Technologies, Santa Clara, CA, United States) (Kennedy and Jones, 2001). 1 mL of wine sample was applied to the Bond Elut C18 OH solid phase extraction cartridges (Agilent Technologies, Santa Clara, CA, United States) to purify wine proanthocyanidins. Eluents were evaporated and resuspended in 1 mL of methanol, and 0.25 mL methanolic extracts were combined with 0.25 mL of phloroglucinolysis reagent (100 g⋅L^–1^ phloroglucinolysis and 20 g⋅L^–1^ ascorbic acid with 0.2 N HCl at methanol). The mixtures were then water bathed at 50°C for 20 min. The reaction was stopped by mixing 200 μL of the sample mixtures with 1 mL of stopping reagent (40 mM aqueous sodium acetate) and then injected into the HPLC. The HPLC column consisted of two Chromolith RP-18e (100 × 4.6 mm^2^) columns serially connected and protected by a guard column with the same material (4 × 4 mm^2^) from EM Science (Gibbstown, NJ, United States). The mobile phase flow rate was 3.0 mL⋅min^–1^. Two mobile phases were used, which included solvent A = 1% aqueous acetic acid (v/v) and solvent B = 1% acetic acid in acetonitrile (v/v). The HPLC flow gradient started with 97% A with 3% B; 82% A, 18% B at 14 min; 20% A, 80% B at 14.01 min; 97% A, 3% B at 16.01 min until 20 min.

All solvents used in this analysis were of HPLC grade, including acetonitrile, methanol, hydrochloric acid, and formic acid purchased from Fisher Scientific (Santa Clara, CA, United States). Standards used for compound identification included malvidin 3-*O*-glucoside, (-)-epicatechin purchased from Extrasynthese (Genay, France). Phloroglucinol was purchased from VWR (Visalia, CA, United States).

### Statistical Analysis

Geostatistical analysis was performed in the R language by using package “gstat” 1.1-6 (Pebesma, 2004). The bulk EC data were filtered by Tukey’s rule to remove outliers either below the first quartile by 1.5 inter-quartile range or above the third quartile by 1.5 inter-quartile range. To further remove the outliers, the data were filtered by the speed that the vehicle was driving, which was between 3.2 km per hour to 8.0 km per hour. Variograms were assessed by “automap” package 1.0-14 (Hiemstra, 2013), and fitted to perform kriging. The soil bulk EC values were extracted from the location of each experimental unit, these values were further used to perform regression analysis. Kriging and *k*-means clustering on plant physiology variables were performed with the R packages “gstat” and “NbClust,” v3.0 (Charrad et al., 2014). Universal kriging was utilized on plant water status because of the existing trend in longitude and latitude. Variograms were assessed by “automap” package 1.0-14 (Hiemstra, 2013), and fitted to perform universal kriging. The vineyard was delineated into two clusters by *k*-means clustering, including Zone 1 with higher water deficit and Zone 2 with lower water deficits. The separation described 78.1% in 2017 of the variability in the plant water status according to the result of between sum of squares/total sum of squares. The resulting maps were organized and displayed by using QGIS software (version 2.14.12, QGIS Development Team). Cluster comparison was analyzed by “raster” package reported as Pearson’s Correlation between two cluster maps (Hijmans et al., 2015).

Data were tested for normality by using Shapiro-Wilk’s test, and subjected to mean separation by using two-way ANOVA with the package “stats” in RStudio (R Foundation for Statistical Computing, Vienna, Austria) (R Core Team, 2019). Significant statistical differences were determined when *p* values acquired from ANOVA were <0.05, and the zones were classified according to Tukey’s honestly significant difference (HSD) test. Regression analysis was performed by SigmaPlot 13.0 (Systat Software Inc., San Jose, CA, United States). Correlation coefficient between variables were calculated in by Pearson’s correlation analysis, and *p*-values were acquired to present the significances of the linear fittings.

### Winemaking Procedures

Vinification was conducted in 2016 and 2017 at the UC Davis Teaching and Research Winery. The grapes were harvested when Zone 1 reached a TSS of 29.88°Bx, 3.92 pH, 5.40 g⋅L^–1^ TA in 2016 and 26.72°Bx, 3.65 pH, 6.53 g⋅L^–1^ TA in 2017, and Zone 2 reached a TSS of 26.32°Bx, 3.75 pH, 6.01 g⋅L^–1^ TA in 2016 and 23.71°Bx, 3.58 pH, 7.22 g⋅L^–1^ TA in 2017. Before dividing the fruits from each zone into three dependent replicate fermentation vessels (200 L each), the grapes were destemmed and crushed once transported into the winery. 50 mg⋅L^–1^ of SO_2_ was added to each vessel to prevent oxidation. Water was added to the musts to balance soluble solid level at 25°Bx due to the highly possible stuck fermentation events may occur based on the high TSS levels. Dilution factors were considered when analyzing the final wine chemical composition. The must samples were inoculated with EC-1118 yeast (Lallemand Lalvin^®^, Montreal, Canada) to initiate the fermentation in jacketed stainless steel tanks controlled by an integrated fermentation control system (T.J fermenters, Cypress Semiconductor Co., San Jose, CA, United States), and two volumes of must were pumped over twice per day by the system. The fermentations were carried at 25°C until the residual sugar contents were below 3 g⋅L^–1^. Malolactic fermentation was initiated with the addition of Viniflora^®^ *Oenococcus oeni* (Chr. Hansen A/S, Hørsholm, Denmark) at 12°C and 60% humidity. The free SO_2_ levels were adjusted to 30 mg⋅L^–1^ after malolactic fermentation completed. Then the wines were sterile filtered and bottled before further chemical analysis. Wine samples were filtered by PTFE membrane filters (diameter: 13 mm, pore size: 0.45 μm, VWR, Seattle, WA, United States) and transferred directly into HPLC vials for anthocyanin analysis.

## Results

### Weather at the Research Site

Between the 2 years of the study, the precipitation amounts were different (Figure 1A). The precipitation amount in the dormant season prior to 2016 was 559.5 mm (from previous harvest date to May as we reported previously; Brillante et al., 2017). However, this amount was 898 mm in the 2016–2017 season. The precipitation during growing seasons in these 2 years were limited, there were only 51.6 mm of precipitation received in 2016 from April to harvest. In 2017, 107 mm of precipitation were received from April to harvest. The research site only received 11.1 mm in 2016 and 15.4 mm in 2017 during the study time in each year from June to harvest. There was a slight difference observed close to harvest (Figure 1B). In 2016, GDD accumulation was 1183°C at harvest (5 October 2016). The GDD accumulation was greater in 2017 at 1220°C by harvest (20 September 2017). The cumulative ET_*o*_ was greater in 2017 compared to 2016 (Figure 1C). At harvest, the cumulative ET_*o*_ was 750 mm in 2016, but it was relatively lower compared to 872.8 mm in 2017.

### Soil Property Assessment

**FIGURE 2:**
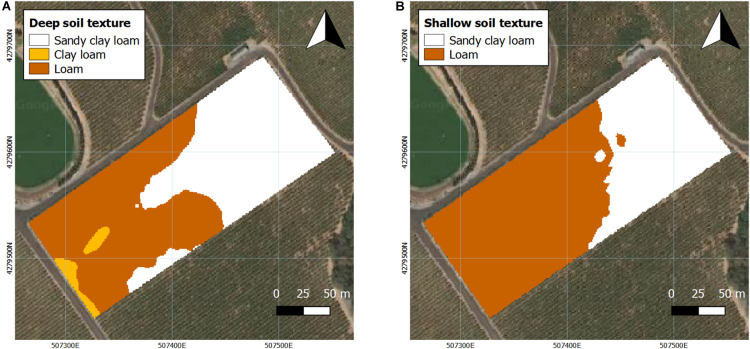
Interpolated projection of soil texture at two depths assessed in 2016 and 2017. **(A)** Deep soil (0.75–1.5 m), **(B)** shallow soil (0–0.75 m). Coordinate system: WGS 1984 UTM Zone 10N.

Soil texture was measured at two different depths (Figure 2). In deep soil, the majority of the westerly section of the vineyard consisted mostly of loam with a small portion of clay loam in the southwestern corner of the vineyard, with the remainder being characterized as sandy clay loam (Figure 2A). In shallow soil, the easterly section of the vineyard mainly was a sandy clay loam with loam comprising the rest of shallow soil of the vineyard (Figure 2B).

**FIGURE 3:**
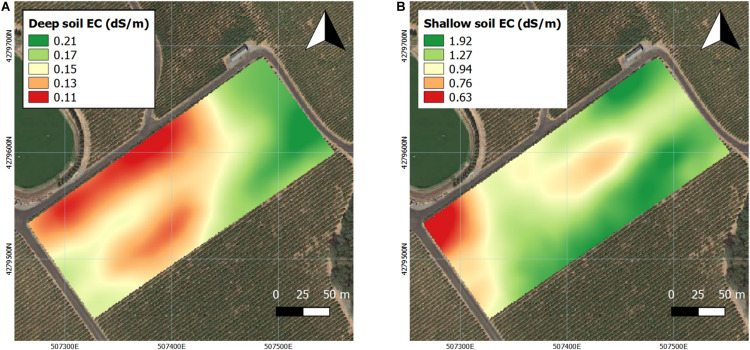
Interpolation soil electrical conductivity (EC) in two depths assessed by EM38 in 2016 and 2017. **(A)** Deep soil (0–1.5 m), **(B)** shallow soil (0–0.75 m). Coordinate system: WGS 1984 UTM Zone 10N.

Soil bulk EC was also assessed at two different depths by proximal sensing in the first season (Figure 3). In deep soil, EC values were lower in the majority of the westerly section of the vineyard (Figure 3A). In shallow soil, EC values were lower in the northwestern corner of the vineyard, and a small portion of the central section also showed lower EC values (Figure 3B).

### Plant Water Status and Leaf Gas Exchange

**FIGURE 4:**
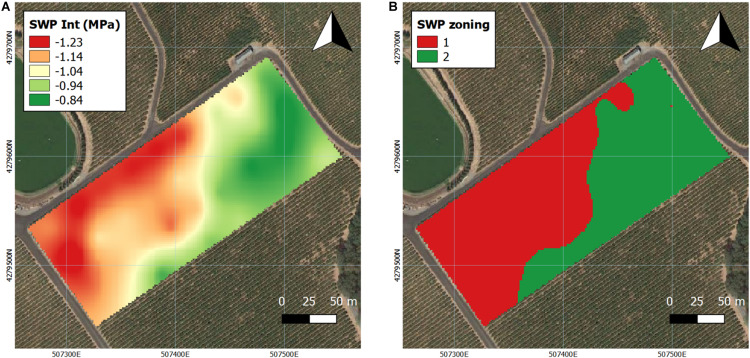
Interpolation maps of plant water status (expressed as stem water potential Ψ_*stem*_) and *k*-means clustering, delineating the vineyard into two zones in 2017. **(A)** Ψ_*stem*_ kriging maps, **(B)** *k*-means clustering maps. Coordinate system: WGS 1984 UTM Zone 10N.

Ψ_*stem*_ was continuously measured as previously reported in 2016 (Brillante et al., 2017) and 2017. Based on the interpolation of Ψ_*stem*_, the trends in the calculated long-term Ψ_*stem*_ integral maps were similar to the trends in the soil bulk EC maps, especially when compared to the deep EC map (Figure 4). Majority of the westerly section of the vineyard had more water stress in 2016 (Brillante et al., 2017) as well as in 2017 (Figure 4A). Then, the interpolation maps of the Ψ_*stem*_ were separated into two zones by *k*-means clustering analysis as Year 1 was reported previously (Brillante et al., 2017). When comparing the two *k*-means clustering maps between 2016 and 2017, there was an 85% similarity according to Pearson’s correlation coefficient between the two maps (Figure 4B). In 2017, the clustering map was 70 and 78% similar to the deep soil and shallow soil texture maps.

**FIGURE 5:**
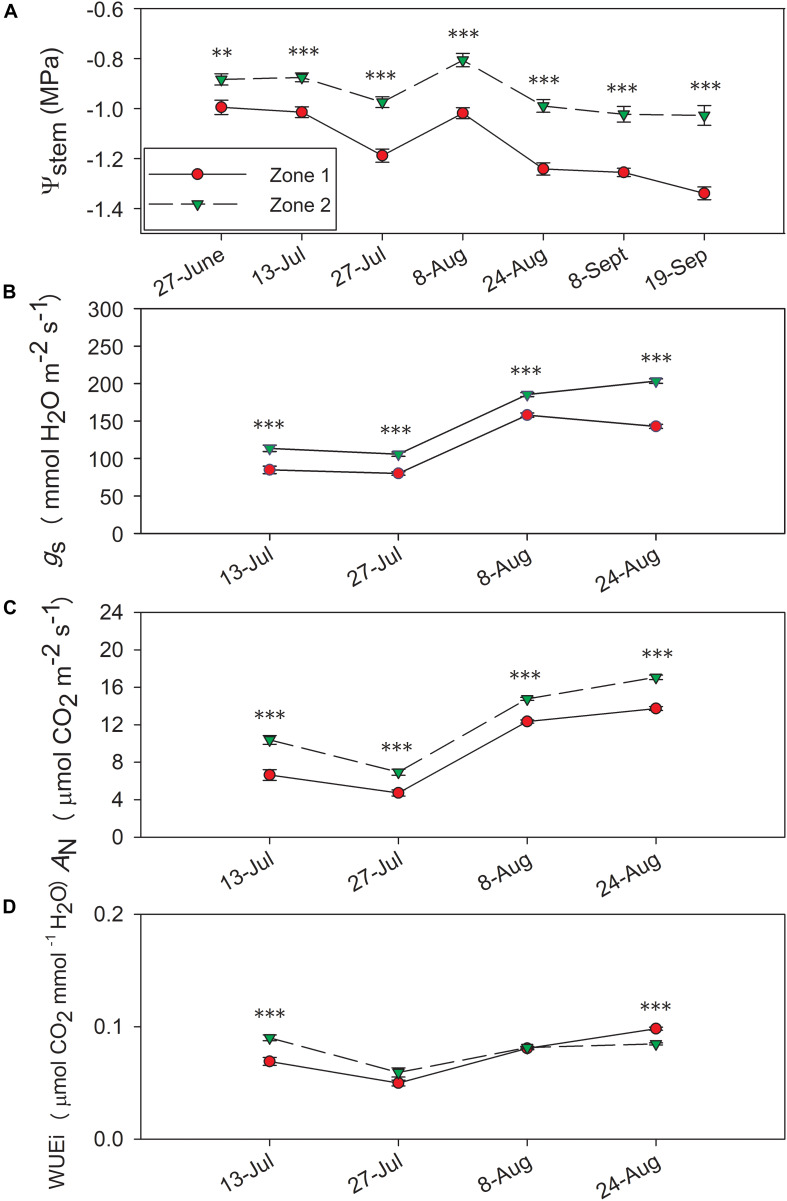
Progression of stem water potential Ψ_*stem*_ and gas exchange between the two water status zones in 2017. **(A)** Ψ_*stem*_, **(B)** stomatal conductance, *g*_*s*_, **(C)** net carbon assimilation, *A*_*N*_, **(D)** intrinsic water use efficiency, WUEi. Error bars represent standard error of the mean. Asterisks represents significant levels *p*: ^∗∗∗^*p* < 0.001, ^∗∗^*p* < 0.01, ^∗^*p* < 0.05.

In 2017, Ψ_*stem*_ were consistently different between the two zones (Figure 5A), where Zone 2 consistently had higher Ψ_*stem*_ than Zone 1. Ψ_*stem*_ values became more negative with the progression of time, and the differences in Ψ_*stem*_ intensified throughout each season as berries reached a more advanced maturity. The differences between two zones ranged from 0.11 MPa on the first measurement day of 27 June to 0.31 MPa on the harvest day of 20 September. Between the two zones, a 0.22 MPa differences in Ψ_*stem*_ integrals were observed in 2017, similar to 0.21 MPa as in 2016 (Brillante et al., 2017).

Leaf gas exchange was measured 2017, where both years showed evident differences between the zones in both *A*_*n*_ and *g*_*s*_ (Figure 5). In 2017, the two zones showed significant differences in *A*_*n*_ and *g*_*s*_ with the highest values observed on 24 August (Figures 5B,C). Conversely, there was no consistent difference in WUE_*i*_ between the two zones in 2017, except Zone 2 showed higher WUEi on 13 July and lower WUEi on 24 August (Figure 5D).

### Yield Components, Must Soluble Solids, pH, and Titratable Acidity

**FIGURE 6:**
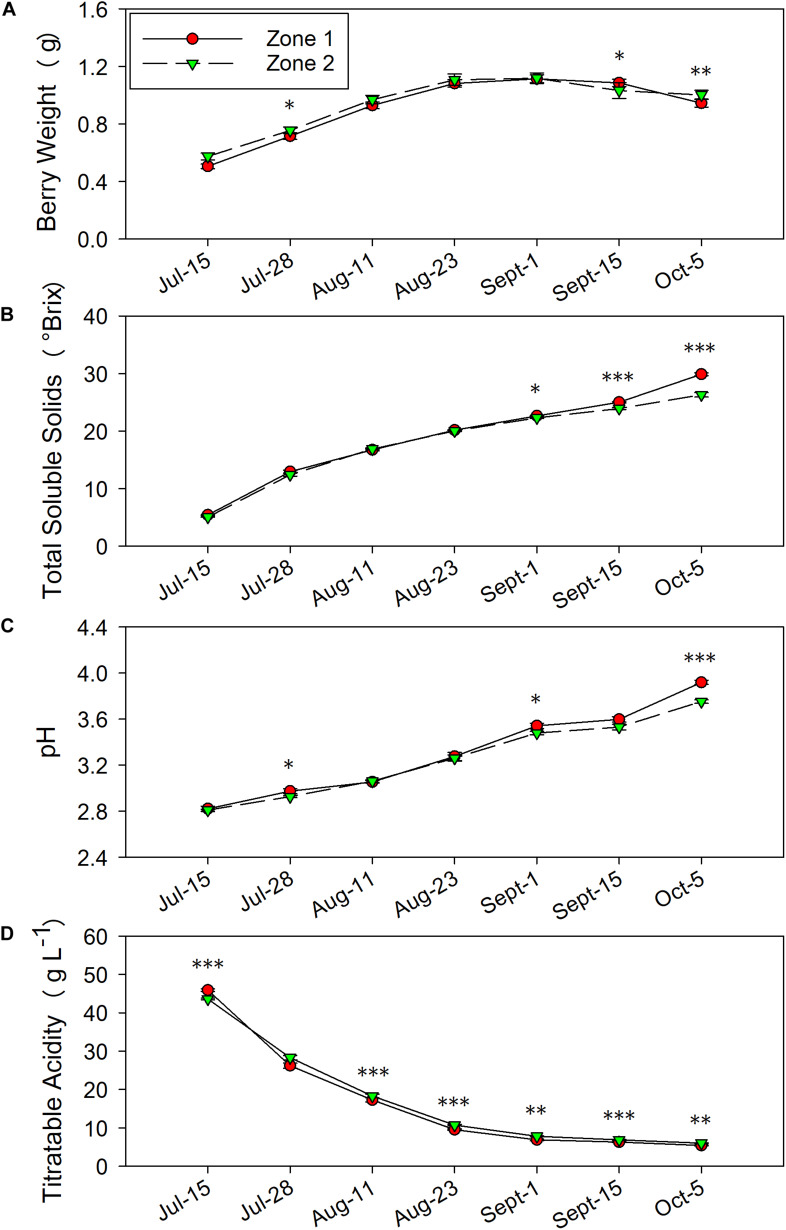
Temporal development of grape berry primary metabolites between the two plant water status zones in 2016. **(A)** Berry weight, **(B)** total soluble solids, **(C)** pH, **(D)** titratable acidity. Error bars represent standard error of the mean. Asterisks represents significant levels *p*: ^∗∗∗^*p* < 0.001, ^∗∗^*p* < 0.01, ^∗^*p* < 0.05.

**FIGURE 7:**
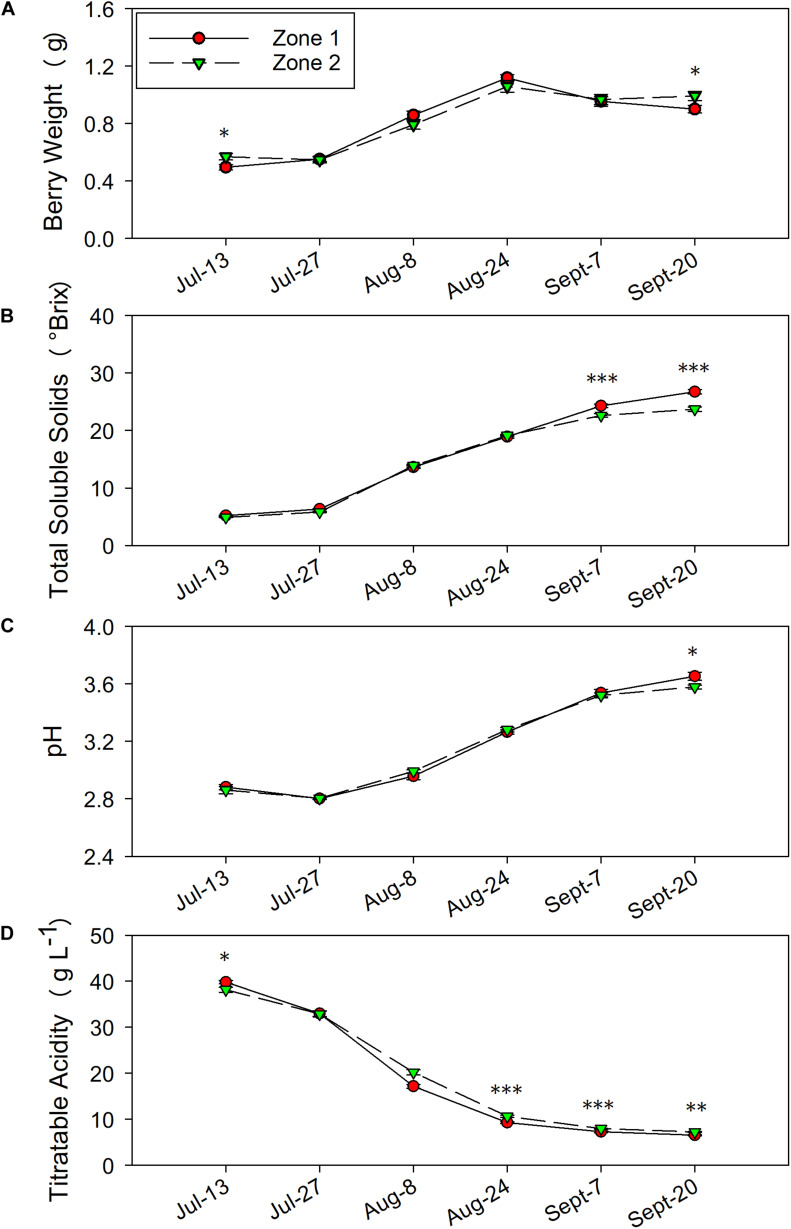
Temporal development of grape berry primary metabolites between the two plant water status zones in 2017. **(A)** Berry weight, **(B)** total soluble solids, **(C)** pH, **(D)** titratable acidity. Error bars represent standard error of the mean. Asterisks represents significant levels *p*: ^∗∗∗^*p* < 0.001, ^∗∗^*p* < 0.01, ^∗^*p* < 0.05.

Components of yield were measured at harvest (Table 1, the harvest data on 5 October 2016 was reported previously in Brillante et al., 2017), and berry primary metabolites were continuously assessed during 2016 and 2017 (Figures 6, 7, the harvest data on 5 October 2016 was reported previously in Brillante et al., 2017). Between the two plant water status zones, there was no differences in yield, berry number, pruning weight, or Ravaz Index. However, there was an effect of experimental year where we measured greater yield and lower pruning weight per vine in Year 2. The only difference observed in yield components was that the berry skin weights were greater in Zone 1 in 2017.

**TABLE 1:** Yield components and berry primary metabolites of Cabernet Sauvignon as separated by water status zoning in Sonoma County, CA in 2016 and 2017^a,b^.

		Yield (tons⋅ha^–1^)	Skin weight (mg)	Berry (no.⋅m^–1^)	Pruning weight (kg⋅vine^–1^)	Ravaz index (kg⋅kg^–1^)	TSS (°Brix) ^c^	pH	TA (g⋅L^–1^) ^d^
2016	Zone 1 ± SE ^w^	7.06 ± 0.48	60.54 ± 2.71	2879.42 ± 157.73	2.08 ± 0.05 ±	2.43 ± 0.26	29.88 ± 0.25 a ^e^	3.92 ± 0.02 a	5.40 ± 0.11 b
	Zone 2 ± SE	6.70 ± 0.77	55.12 ± 2.76	2529.03 ± 230.84	2.07 ± 0.14	2.31 ± 0.45	26.32 ± 0.38 b	3.75 ± 0.01 b	6.01 ± 0.12 a
	*p*-value	ns	ns	ns	ns	ns	0.027	<0.0001	0.001
2017	Zone 1 ± SE	13.66 ± 0.66	62.25 ± 1.80 a	5997.40 ± 341.15	1.66 ± 0.07	5.88 ± 0.37	26.72 ± 0.39 a	3.65 ± 0.03 a	6.53 ± 0.12 b
	Zone 2 ± SE	11.68 ± 1.45	52.98 ± 2.04 b	4408.16 ± 434.27	1.70 ± 0.14	4.92 ± 1.02	23.71 ± 0.40 b	3.58 ± 0.01 b	7.22 ± 0.10 a
	*p*-value	ns	0.002	ns	ns	ns	<0.0001	0.024	<0.0001
Year		<0.0001	< 0.0001	<0.0001	0.002	0.0001	<0.0001	< 0.0001	<0.0001
Year × Zoning		ns	ns	ns	ns	ns	ns	ns	ns

*^*a*^n = 35, ns: not significant, the data for 2016 in this table was partially published in Brillante et al. (2017), courtesy of American Chemical Society. ^*b*^Except skin weight, components of yield were expressed as numbers or weight per meter of vineyard row. ^*c*^TSS, total soluble solids; TA, titratable acidity. ^*d*^Zone 1: lower plant water status zone, Zone 2: higher plant water status zone. Numbers in the column were expressed as their means ± standard error of the mean. ^*e*^Different letters indicate significant mean separation according to Tukey’s HSD test (p < 0.05).*

The berry primary metabolites were different between the two zones in both years of the study. In 2016, berry weights were greater in Zone 2 on 28 July and 5 October when the fruits were harvested (Figure 6A). Zone 1 showed higher berry weights on 15 September. When the irrigation was stopped at veraison, TSS were higher in Zone 1 compared to Zone 2, which was measured on 1 September, 15 September, and 5 October (Figure 6B). At harvest, the fruits in Zone 1 reached a TSS of 29.9°Bx, while the ones in Zone 2 reached 26.3°Bx. The juice pH showed a similar result with TSS, where Zone 1 had higher pH in the last 3 months before harvest, except there was no difference shown on 15 September (Figure 6C). Berry TA was consistently higher in Zone 2 on all measured dates except 28 July (Figure 6D). At harvest, Zone 2 had 6.0 g⋅L^–1^ of TA, Zone 1 had 5.4 g⋅L^–1^.

In 2017, the differences in TSS, pH and TA were similar to 2016. Berry weights were higher in Zone 2 on 13 July and at harvest on 20 September (Figure 7A). The TSS increased more rapidly in Zone 1 close to harvest on 7 September and 20 September (Figure 7B) when compared to Zone 2. At harvest, TSS values were slightly lower than 2016 due to an earlier harvest time, where Zone 1 reached a TSS of 26.7°Bx, while Zone 2 reached 23.7°Bx (Table 1). The juice pH was higher in Zone 1 than Zone 2 at harvest as well (Figure 7C). Similar to 2016, the TA in the two zones was consistently different where Zone 2 had higher TA than Zone 1 on starting on 24 August until harvest (Figure 7D). At harvest, Zone 2 had 7.2 g⋅L^–1^ of TA, Zone 1 had 6.5 g⋅L^–1^.

### Berry Skin Anthocyanins at Harvest

**FIGURE 8:**
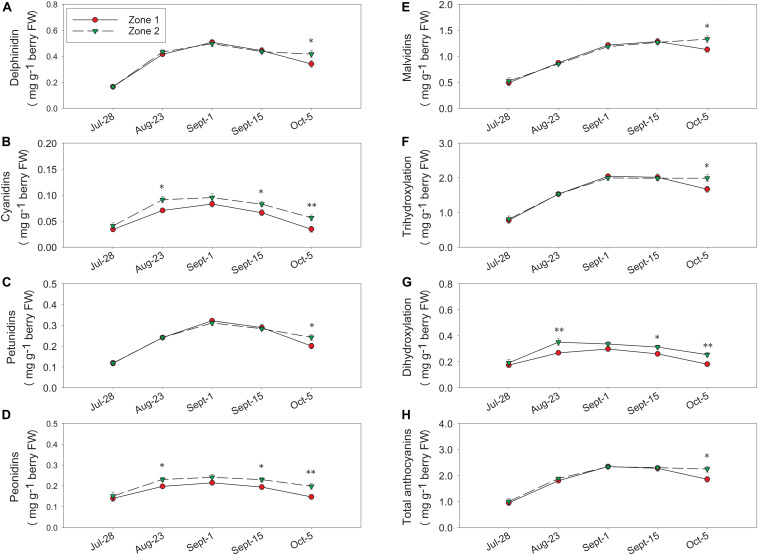
Temporal development of grape berry anthocyanins between the two plant water status zones in 2016. **(A)** Delphinidins, **(B)** cyanidins, **(C)** petunidins, **(D)** peonidins, **(E)** malvidins, **(F)** tri-hydroxylation, **(G)** di-hydroxylation, **(H)** total anthocyanins. Error bars represent standard error of the mean. Asterisks represents significant levels *p*: ^∗∗∗^*p* < 0.001, ^∗∗^*p* < 0.01, ^∗^*p* < 0.05.

Berry skin anthocyanins were different between the two zones in 2016 (the harvest data on 5 October 2016 was partially reported previously in Brillante et al., 2017). Total delphinidins, petunidins, malvidins, and the sum of them as tri-hydroxylated anthocyanins were all higher in Zone 2 than Zone 1 (Figures 8A,C,E,F,H). Total cyanidins, peonidins, and the sum of them as di-hydroxylated anthocyanins were greater in Zone 2 on 23 August, 15 September, and at harvest (Figures 8B,D,G). Total skin anthocyanins were 2.2 mg per g of berry fresh weight (FW) in Zone 2 which was higher than the 1.85 mg measured in Zone 1 (Table 2).

**TABLE 2:** Grape skin anthocyanins of Cabernet Sauvignon as separated by water status zoning in Sonoma County, CA in 2016 and 2017^a,b^.

		Delphinidin (mg⋅g^–1^)	Cyanidin (mg⋅g^–1^)	Petunidin (mg⋅g^–1^)	Peonidin (mg⋅g^–1^)	Malvidin (mg⋅g^–1^)	Tri-OH (mg⋅g^–1^) ^c^	Di-OH (mg⋅g^–1^)	Total anthocyanins (mg⋅g^–1^)
2016	Zone 1 ± SE ^d^	0.34 ± 0.03	0.03 ± 0.01 b ^e^	0.20 ± 0.01 b	0.15 ± 0.01 b	1.13 ± 0.05 b	1.67 ± 0.09 b	0.18 ± 0.02 b	1.85 ± 0.10 b
	Zone 2 ± SE	0.42 ± 0.03	0.06 ± 0.01 a	0.24 ± 0.01 a	0.20 ± 0.01 a	1.33 ± 0.06 a	1.99 ± 0.10 a	0.25 ± 0.02 a	2.24 ± 0.11 a
	*p*-value	ns	0.010	0.039	0.007	0.018	0.027	0.007	0.016
2017	Zone 1 ± SE	0.34 ± 0.01	0.05 ± 0.00	0.24 ± 0.01	0.17 ± 0.01	1.33 ± 0.04 a	1.91 ± 0.05 a	0.21 ± 0.01	2.12 ± 0.06 a
	Zone 2 ± SE	0.33 ± 0.02	0.05 ± 0.01	0.23 ± 0.01	0.18 ± 0.01	1.14 ± 0.02 b	1.70 ± 0.05 b	0.24 ± 0.02	1.94 ± 0.06 b
	*p*-value	ns	ns	0.002	ns	0.000	0.005	ns	0.000
Year		0.018	<0.0001	0.001	<0.0001	ns	ns	0.0001	ns
Year × Zoning		ns	ns	ns	ns	0.012	0.021	ns	0.020

*^*a*^n = 35, ns: not significant, the data for 2016 in this table was partially published in Brillante et al. (2017), courtesy of American Chemical Society. ^*b*^All compounds are expressed in the unit of mg per g of berry fresh weight. All anthocyanins comprised of their -glucoside, -acetyl glucoside, and -coumaroyl glucoside derivatives. ^*c*^-OH, hydroxylation. Tri-OH included delphinidin, petunidin and malvidin, di-OH included cyanidin and peonidin. ^*d*^Zone 1: lower plant water status zone, Zone 2: higher plant water status zone. Numbers in the column were expressed as their means ± standard error of the mean. ^*e*^Different letters indicate significant mean separation according to Tukey’s HSD test (p < 0.05)*.

**FIGURE 9:**
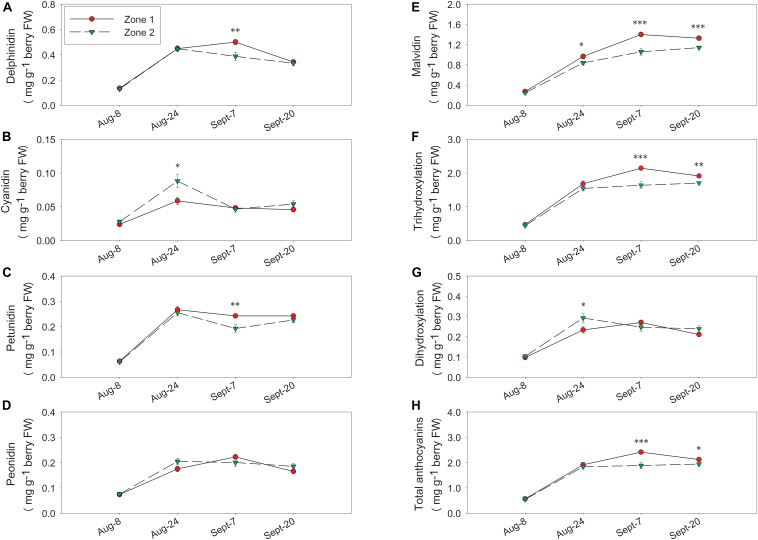
Temporal development of grape berry anthocyanins between the two water status zones in 2017. **(A)** Delphinidins, **(B)** cyanidins, **(C)** petunidins, **(D)** peonidins, **(E)** malvidins, **(F)** tri-hydroxylation, **(G)** di-hydroxylation, **(H)** total anthocyanins. Error bars represent standard error of the mean. Asterisks represents significant levels *p*: ^∗∗∗^*p* < 0.001, ^∗∗^*p* < 0.01, ^∗^*p* < 0.05.

In 2017, there were no differences between the two zones in delphinidin, cyanidin, petunidin, or peonidin at harvest (Figures 9A–D). Zone 1 had higher malvidins from 24 August until harvest, and tri-hydroxylated anthocyanins, total anthocyanins from 7 September until harvest (Figures 9E,F,H). Conversely, total malvidins, tri-hydroxylated anthocyanins, and total anthocyanins were higher in Zone 1 at harvest (Table 2). In Zone 2, we measured higher cyanidins and di-hydroxylated anthocyanins on 24 August (Figures 9B,G), and that was the only date Zone 2 had higher concentrations in any of these derivatives.

**FIGURE 10:**
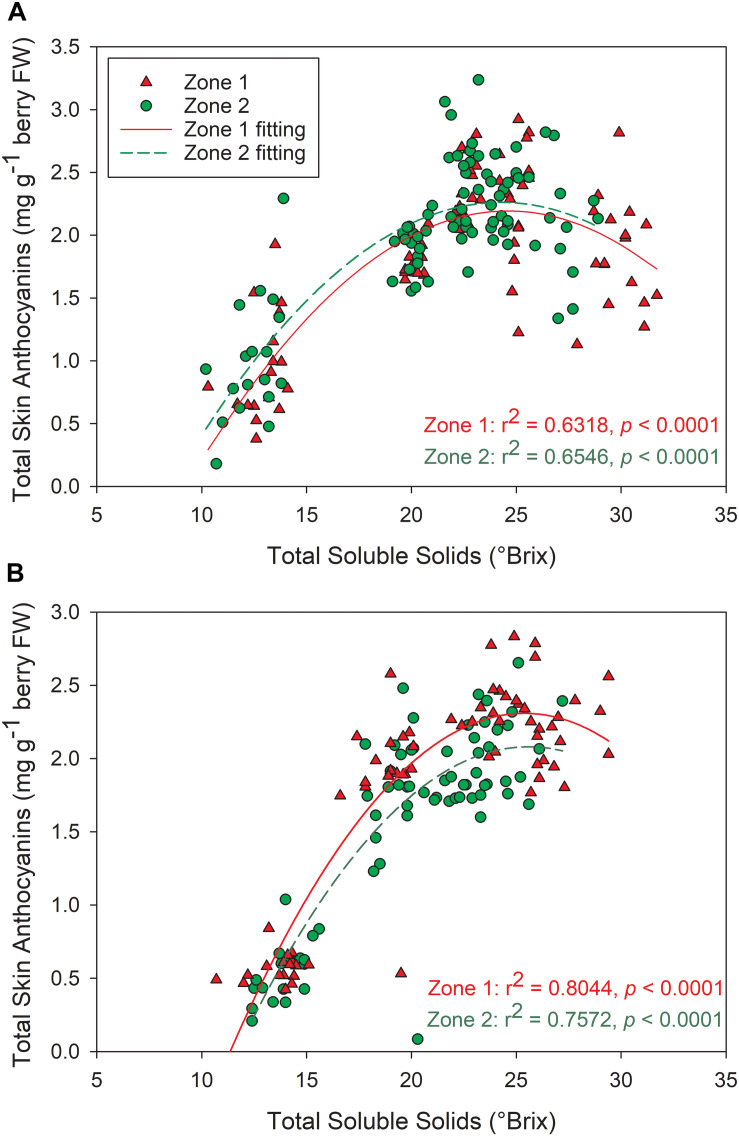
Relationships between grape berry total soluble solids and total skin anthocyanins between the two plant water status zones in **(A)** 2016, **(B)** 2017. ^∗∗∗^*p* < 0.001, ^∗∗^*p* < 0.01, ^∗^*p* < 0.05.

The temporal relationships between TSS and berry skin anthocyanins were investigated in both years (Figure 10). In both years, skin anthocyanins increased with the accumulation of TSS at first. In 2016, berry anthocyanins of Zone 1 had a significant decline in skin anthocyanins after 25°Bx TSS, resulting a lower concentration when compared to Zone 2 (Figure 10A). Conversely, the second season consistently showed greater anthocyanin concentration in Zone 1 than Zone 2 (Figure 10B). However, Zone 1 showed a more rapid decline after around 25°Bx TSS, and the skin anthocyanins were similar in values with Zone 2.

### Wine Flavonoids

Wine-free anthocyanins and proanthocyanidins were assessed in both years. For anthocyanins, Zone 2 had higher concentrations of all derivatives in 2016, including tri-, di- hydroxylated, and total anthocyanins (Table 3). All the compounds were more than two times greater than Zone 1. However, there was no difference observed in any of these compounds in 2017. The overall concentrations of all these compounds were greater in 2017 than 2016.

**TABLE 3:** Free anthocyanins in Cabernet Sauvignon wines as separated by water status zoning in Sonoma County, CA in 2016 and 2017^a,b^.

		Delphinidin (mg⋅L^–1^)	Cyanidin (mg⋅L^–1^)	Petunidin (mg⋅L^–1^)	Peonidin (mg⋅L^–1^)	Malvidin (mg⋅L^–1^)	Tri-OH (mg⋅L^–1^)^c^	Di-OH (mg⋅L^–1^)	Total anthocyanins (mg⋅L^–1^)
2016	Zone 1 ± SE ^d^	21.36 ± 0.36 b ^e^	0.80 ± 0.01 b	21.01 ± 0.36 b	8.58 ± 0.13 b	163.69 ± 2.76 b	206.06 ± 3.45 b	9.38 ± 0.14 b	215.44 ± 3.59 b
	Zone 2 ± SE	51.35 ± 1.98 a	2.37 ± 0.09 a	45.45 ± 1.54 a	21.33 ± 0.75 a	350.23 ± 9.73 a	447.03 ± 13.25 a	27.53 ± 0.97 a	470.73 ± 14.08 a
	*p*-value	<0.0001	< 0.0001	<0.0001	< 0.0001	<0.0001	< 0.0001	<0.0001	< 0.0001
2017	Zone 1 ± SE	75.88 ± 1.46	4.94 ± 0.08	53.97 ± 0.92	33.42 ± 0.47	614.15 ± 8.48	743.99 ± 10.01	38.37 ± 0.54	782.36 ± 10.39
	Zone 2 ± SE	76.77 ± 1.65	4.82 ± 0.12	53.94 ± 0.99	33.40 ± 0.58	597.09 ± 9.75	727.80 ± 11.77	38.22 ± 0.69	766.02 ± 12.28
	*p*-value	Ns	ns	0.002	ns	0.000	0.005	ns	ns
Year		<0.0001	< 0.0001	<0.0001	< 0.0001	<0.0001	< 0.0001	<0.0001	< 0.0001
Year × Zoning		<0.0001	< 0.0001	<0.0001	< 0.0001	<0.0001	< 0.0001	<0.0001	< 0.0001

*^*a*^n = 9, ns: not significant. ^*b*^All compounds are expressed in the unit of mg per L. All anthocyanins comprised of their -glucoside, -acetyl glucoside, and -coumaroyl glucoside derivatives. ^*c*^ -OH, hydroxylation. Tri-OH included delphinidin, petunidin and malvidin, di-OH included cyanidin and peonidin. ^*d*^Zone 1: lower plant water status zone, Zone 2: higher plant water status zone. Numbers in the column were expressed as their means ± standard error of the mean. ^*e*^Different letters indicate significant mean separation according to Tukey’s HSD test (p < 0.05)*.

For proanthocyanidins, similar results were observed (Table 4). In 2016, all the extension and terminal subunits were higher in Zone 2 than Zone 1. The amount of total proanthocyanidins were also higher in Zone 2. In 2017, however, there was no difference observed in any of these subunits or total proanthocyanidins. Again, the second season showed greater concentrations in all of these compounds compared to the first season. Neither year showed difference in mDP between the two zones.

**TABLE 4:** Wine proanthocyanidin subunits of Cabernet Sauvignon as separated by water status zoning in Sonoma County, CA in 2016 and 2017^a,b^.

		Extension subunits (mg⋅L^–1^)				Terminal subunits (mg⋅L^–1^)		Total proanthocyanins (mg⋅L^–1^)	mDP ^c^
			
		EGC ^c^	C ^c^	EC ^c^	ECG ^c^	C ^c^	EC ^c^		
2016	Zone 1 ± SE ^d^	75.07 ± 7.08 b^e^	10.07 ± 0.36 b	123.05 ± 9.13 b	2.73 ± 0.16 b	53.21 ± 1.57 b	21.39 ± 0.86 b	285.52 ± 18.14 b	3.73 ± 0.12
	Zone 2 ± SE	107.94 ± 10.05 a	14.14 ± 0.90 a	174.11 ± 12.40 a	3.70 ± 0.10 a	78.21 ± 1.97 a	38.75 ± 0.90 a	416.85 ± 22.70 a	3.51 ± 0.14
	*p-*value	0.017	0.001	0.004	<0.0001	< 0.0001	<0.0001	< 0.0001	ns
2017	Zone 1 ± SE	453.89 ± 40.68	33.83 ± 2.07	611.69 ± 46.57	9.18 ± 0.78	113.95 ± 9.33	106.00 ± 9.25	1328.55 ± 86.78	5.90 ± 0.38
	Zone 2 ± SE	519.56 ± 68.02	38.77 ± 4.42	690.87 ± 80.86	14.21 ± 2.67	139.08 ± 18.43	127.54 ± 6.85	1530.03 ± 169.24	5.59 ± 0.41
	*p-*value	Ns	ns	ns	ns	ns	ns	ns	ns
Year		<0.0001	< 0.0001	<0.0001	<0.0001	<0.0001	<0.0001	<0.0001	< 0.0001
Year × Zoning		Ns	ns	ns	0.012	ns	ns	ns	ns

*^*a*^n = 9, ns: not significant. ^*b*^All compounds are expressed in the unit of mg per L. ^*c*^C, (+)-catechin; EC, (-)-epicatechin; ECG, (-)-epicatechin-3-O-gallate; EGC, (-)-epigallocatechin; mDP: mean degree of polymerization. mDP was calculated as the ratio of total proanthocyanidins to the terminal subunits. ^*d*^Zone 1: lower plant water status zone, Zone 2: higher plant water status zone. Numbers in the column were expressed as their means ± standard error of the mean. ^*e*^Different letters indicate significant mean separation according to Tukey’s HSD test (p < 0.05).*

### Linking Soil to Grapevine Physiology

The relationships between soil bulk EC and whole grapevine physiology were investigated (Table 5). Soil bulk EC values at both depths increased when Ψ_*stem*_ became more positive, and soil bulk EC and Ψ_*stem*_ were significantly correlated in both seasons. The relationships between soil bulk EC and TSS reflected the relationships between soil bulk EC and Ψ_*stem*_. They showed significant relations with each other in both years. In 2016, Ψ_*stem*_ showed a positive relationship with berry weight at harvest. No significant correlation was observed between soil bulk EC and berry weight. However, shallow soil bulk EC showed a positive correlation with berry weight besides Ψ_*stem*_ in 2017. Berry skin weight and total anthocyanins did not have any significant relationships with neither Ψ_*stem*_ nor soil bulk EC in 2017. In the same year, both berry skin weight and total anthocyanins were positively correlated with Ψ_*stem*_, deep EC, and shallow EC. No parameters related to final yield except deep EC had a positive relationship with it in 2017.

**TABLE 5:** Correlation matrices, values were expressed in Pearson Correlation values of “r” in a commercial Cabernet Sauvignon vineyard in Sonoma County, CA in 2016 and 2017^a,b^.

		SWP Int	Deep EC	Shallow EC	TSS	Berry weight	Skin weight	TSA	Yield
2016	SWP Int		0.6***^c^	0.68***	−0.81***	0.46**	0.22	0.24	0.19
	Deep EC	0.6***		0.5**	−0.69***	0.03	0.07	0.24	0.17
	Shallow EC	0.68***	0.5**		−0.57***	0.25	0.22	0.27	0.06
2017	SWP Int		0.73***	0.59***	−0.83***	0.49**	0.49**	0.6***	0.20
	Deep EC	0.73***		0.5**	−0.68***	0.18	0.35*	0.53**	0.41*
	Shallow EC	0.59***	0.5**		−0.67***	0.51**	0.55**	0.39*	0.02

^*a*^df = 33. ^*b*^SWP Int: stem water potential integrals, EC: soil bulk electrical conductivity, TSS: total soluble solids, TSA: total skin anthocyanins. ^*c*^Asterisks represents significant levels p: ***p < 0.001, **p < 0.01, *p < 0.05.

## Discussion

### Soil Bulk EC and Plant Water Status Spatial Relationships

Site topography influences plant water status (Brillante et al., 2017). In our previous work, we reported that absolute elevation of a vineyard was directly related to Y_*stem*_. The correlation between Y_*stem*_ and elevation was significant and negative, indicating that the Y_*stem*_ would be lower when the elevation was higher. When soil moisture was model as wetness index, it indicated a negative and significant relationship with Y_*stem*_ but the relationship was not linear. In our previous work, we were unable to deduce a significant relationship between site topography variables such as absolute elevation and berry chemistry (Brillante et al., 2017). Bramley et al. (2011a) showed that soil bulk EC was directly related to soil clay content, which was contradictory to our findings. We attributed this discrepancy to the relatively stable soil texture throughout the season or even several seasons. On the other hand, the effect of soil water content might be the major factor to influence plant development during the season. The soil texture and soil bulk EC sensing analysis conducted in this study were able to explain the variability in plant water status that the site topography could not. Soil texture and soil bulk EC can be related to spatial differences in soil water availability (Tramontini et al., 2013). Specifically, soil texture is a determinant of soil water holding capacity, hence affecting the amount of water available to the plants. In our study, the western section of the vineyard had greater loam proportion, where the grapevines were experiencing more severe water deficits (Brillante et al., 2017). The eastern section had more sandy soil in both deep and shallow soil, where the grapevines were under less severe water deficits. Our findings are corroborated with previous work, where clay soil would lead to less plant available water, although clay soil had higher water holding capacity than sandy soil (Tramontini et al., 2013). Furthermore, Cabernet Sauvignon grapevines grown in clay soil would result in lower *g*_*s*_ and *A*_*n*_ compared to grapevines grown in soils that had higher proportion of sandy soils (Yu and Kurtural, 2020).

There was evident variability in soil bulk EC in this study. Previous studies reported that when soil bulk EC was proximally sensed, it was closely related to soil water content (Bittelli, 2011; Brillante et al., 2015). We found that soil bulk EC was consistently and directly related to long-term Ψ_*stem*_ over the course of our study. Our findings are corroborated by previous works (Rodríguez-Pérez et al., 2011; Brillante et al., 2014), where higher soil bulk EC values corresponded to higher soil water content. Previous studies suggested that the relationship between soil water content and soil bulk EC was soil-specific, and needed to include soil chemical and physical properties to explain variability and plant water status (Morari et al., 2009; Brillante et al., 2016b). Due to the limited amount of water put into wine grape vineyards, soil water content would be the major factor affecting soil electrical properties rather than the residual salinity after water evaporation from soil. The significant relationship between soil bulk EC and Ψ_*stem*_ in this study agreed with previous studies, indicating the possibility of soil bulk EC sensing being used to assess plant water status (Bramley et al., 2011a; Rodríguez-Pérez et al., 2011). Moreover, in our study, the spatial variability in grapevine physiology reflected the variability in soil bulk EC very well when assessed by proximal sensing. Due to the relationship of soil bulk EC on the amount of available water to plants reported in previous research (Rodríguez-Pérez et al., 2011; Brillante et al., 2014), this approach had been utilized to identify the variability in the plant physiology based on the soil sensing technologies and apply targeted management strategies (Bramley et al., 2011a), and our study provided more evidence toward the feasibility of it.

The variability we measured proximally in soil characteristics was reflected in plant water status and leaf gas exchange in our study. Previous research had reported that variable soil characteristics in space would cause spatial variations in plant water status (Brillante et al., 2016a). Although the precipitation amounts were vastly different between the two dormant seasons, the uniformly scheduled irrigation did not ameliorate the natural spatial variability in plant water status induced by soil properties. On the contrary, the separations in plant water status and leaf gas exchange were already significant even before the irrigation ceased after veraison. This proved that the spatial variability in the soil dominated the accessibility of the available soil water toward the plant, and made the spatial variability expressed in the grapevine. Our results in the second year corroborated those of the first year, showing that the separation in both plant water status and leaf gas exchange between the two zones were consistent.

Leaf gas exchange was closely related to plant water status, and this relationship was shown in previous research (Costa et al., 2012). The relationships between leaf gas exchange and plant water status were evident in our study, where a higher Ψ_*stem*_ would promote a greater stomatal conductance to increase carbon assimilation capacity and decrease intrinsic water use efficiency. In our study, the lowest Ψ_*stem*_ we observed were around harvest with Ψ_*stem*_ of -1.6 MPa and *g*_*s*_ of around 50 mmol H_2_O m^–2^⋅s^–1^, which were not severe enough to impair berry ripening although the photosynthetic activities were still affected. Overall, the *g*_*s*_ and *A*_*N*_ reached the maximum values at veraison and declined with decreasing plant water status and leaf age toward the end of the season. This further affirmed that the continuous water deficits during the growing season, especially being more pronounced after irrigation was ended after veraison, would reduce stomatal conductance. The water deficits would act as passive hydraulic signals or active hormonal signals with the upregulation in abscisic acid (ABA) synthesis to limit plant photosynthetic activities, hence lower *g*_*s*_ and *A*_*N*_ values (Costa et al., 2012; Tombesi et al., 2015).

### Components of Yield

According to the previous research, components of yield may be affected by plant water status, where higher water deficits would result in reductions of yield, berry skin weight, and berry weight (Williams, 2010; Korkutal et al., 2011; Santesteban et al., 2011). In our study, we observed constant separation in plant water status after veraison. However, there was no difference shown in cluster number, yield, berry number, or pruning weight. The only difference measured in yield components was that berry skin weight was higher in Zone 1 in the second season. Early season water deficit irrigation (prior to veraison) had higher probability to decrease yield than later season water deficit irrigation (post-veraison to harvest). However, a season-long water deficit irrigation would have the lowest yield even despite the season-long water deficit irrigation regime applying double amount of water than the other regimes (Tarara et al., 2011). Some other studies did not have the same results, as early water deficit irrigation did not show significant influences on yield compared to late water deficit irrigation (Intrigliolo and Castel, 2010; Intrigliolo et al., 2012). Another possible explanation was that Zone 1 had greater water amount held in the soil due to the higher clay content. The clay soil with higher water-holding capacity had a better water status at the early season compared to Zone 2, even though the sandy soil in Zone 2 would benefit the plant growth with irrigation when the season progressed (Tramontini et al., 2013). The later season water deficit was exacerbated in Zone 1 due to its higher clay content, causing Zone 1 lost the benefits from the high water status in the early season, and eventually had similar yield components with Zone 2 at harvest. In our work, we did not see any evidence of Ravaz index being affected by spatial variability of plant water status. These results were corroborated by Terry and Kurtural when grapevine cultivar ‘Syrah’ was exposed to post-veraison water deficits in comparable severity of -1.4 MPa (Terry and Kurtural, 2011).

### Must-Soluble Solids, pH, and Titratable Acidity

Water deficits affect advancement of grape berry maturity, they promote TSS accumulation and TA degradation in grape berries (Basile et al., 2011; Williams, 2012). Two factors contributed to these differences between the two zones. First, a greater water deficit advanced the berry maturation, leading to a higher TSS and lower TA (Escalona et al., 2015). Second, berry dehydration may have occurred and the TSS concentration increased in the berries. In our study, smaller berries were observed in Zone 1, which can confirm the berry dehydration could have led to higher TSS in Zone 1. As for berry TA, one study showed that grape organic acids biodegradation would be faster with more solar radiation and higher temperature (Cholet et al., 2016). Although the acid degradation was not related to water deficits, like mentioned above, water deficits would limit the grapevines’ ability to regulate temperature (Tombesi et al., 2015). Thus, water deficits could promote the organic acid degradation and this effect was observed in this study.

### Berry Skin and Wine Flavonoids

Mild water deficits increased the flavonoid content and concentration of red-skinned grape berry due to the upregulation in flavonoid synthesis and the advancement of berry dehydration during growing season (Castellarin et al., 2007a; Bondada and Shutthanandan, 2012). A positive relationship was noticed between soil bulk EC and total skin anthocyanins in 2017 at both depths of soil bulk EC measurements. A more prolonged severe water deficit would lead to deleterious stomatal and temperature regulation and eventually resulted in flavonoid degradation, specifically anthocyanins (Movahed et al., 2016). This was a plausible explanation for the non-significant relationship between soil bulk EC and total skin anthocyanins in 2016, wherein harvest took place at higher soluble solids and Zone 1 berry skin anthocyanins were presumably in decline. Furthermore, the berry weights were higher in Zone 2, which was similar to the observations in our previous work (Martínez-Lüscher et al., 2017), indicating there was less berry dehydration. Thus, the higher anthocyanins in Zone 2 was mainly due to the upregulation in anthocyanins other than anthocyanins degradation. These effects were also observed in the wines of 2016, where Zone 2 had higher anthocyanin concentrations. However, in the second season, the differences in berry skin anthocyanins at harvest did not carry over into the wines. We contributed this to the more advanced berry maturity levels at harvest in the first season, the skin cell walls could have become more porous during ripening and increased the extractability of flavonoid compounds (Bindon et al., 2014). With relatively greater amounts of flavonoids extracted, there was a higher chance to pass on the separations of anthocyanins from the berries to the wines.

Grape berry skin proanthocyanidins are less sensitive toward water deficits than anthocyanins (Castellarin et al., 2007a; Cáceres-Mella et al., 2017). Nevertheless, their biosynthesis and concentration may be modified by water deficits (Ollé et al., 2011; Cáceres-Mella et al., 2017). In 2016, wine total proanthocyanidins and all the subunits were greater in Zone 2. These differences were not observed in the second season. We attributed this lack of consistency in proanthocyanidin disparities between the two zones to the more advanced maturity of the berries were harvested in 2016 than in 2017. We suggest that similar to skin anthocyanins, the more advanced berry maturity in 2016 could have promoted the proanthocyanidin extractability in the skin tissues (Bindon et al., 2014), which may augment the separations in the concentration of all the subunits between the two zones.

## Conclusion

Our work provided evidence of the connection between soil bulk EC sensing and whole plant physiology, and the effects of which then cascaded to berry and wine chemistry. We presented that soil bulk EC in vineyard systems affected plant water status. The clusters of plants with similar water status may comprise zones of similar physiological behavior due to these inherent differences from different plant water status, and the discrepancies in plant water status resulted in cascading effects on berry chemistry. In conclusion, our work provides fundamental knowledge about the applicability of soil bulk EC sensing in the vineyards, and its potential directional utilization by connecting proximal sensing to spatial distribution of whole-plant physiological performance together with berry and wine chemistry.

## Data Availability Statement

The raw data supporting the conclusions of this article will be made available by the authors, without undue reservation, to any qualified researcher.

## Author Contributions

SK acquired the funding and designed the trial. LB, RY, and JM-L executed the trial. RY made the wine, analyzed the metabolites, and wrote the first version of the manuscript. All authors contributed to the final version and approved.

## Conflict of Interest

The authors declare that the research was conducted in the absence of any commercial or financial relationships that could be construed as a potential conflict of interest.
